# Spatially heterogeneous ionic remodelling promotes wavefront instability in simulated atrial tissue

**DOI:** 10.3389/fnetp.2026.1849952

**Published:** 2026-07-30

**Authors:** Luke O’Loughlin, Dhani Dharmaprani, Anand Ganesan, Lewis Mitchell

**Affiliations:** 1 School of Mathematical Sciences, Adelaide University, Adelaide, SA, Australia; 2 College of Science and Engineering, Flinders University, Adelaide, SA, Australia; 3 College of Medicine and Public Health, Flinders University, Adelaide, SA, Australia

**Keywords:** atrial fibrillation, computational modelling, heterogeneity, ising model, network physiology, remodelling

## Abstract

Electrical remodelling is often seen as a key determinant in the development of atrial fibrillation. From a computational electrical modelling point of view, remodelling is usually incorporated by a global (within a specific atrial region) change of model parameters, e.g., ionic conductances and calcium handling properties. Given the spatially heterogeneous nature of cardiac tissue, one may expect that remodelling develops in a non-uniform manner. Computational models sometimes reflect this when fibrotic regions are considered; however, in the case of spatially heterogeneous electrical remodelling the literature is much sparser. Here we demonstrate that a spatially heterogeneous mixture of remodelled and non-remodelled regions can cause a destabilizing effect in simulated tissue paced at a sufficiently high frequency, indicating a potential mechanism for the initiation of fibrillatory behaviour. In particular, we use an established model of human atrial action potential with parameters based on sinus rhythm and chronic atrial fibrillation, and we distribute these two possible states according to realisations of the 2-dimensional Ising model. We show that there is a window of pacing frequencies where turbulent propagation behaviour (via the break up of incoming wavefronts) is sustained, and that a long turbulent transient can be sustained when adapting to fast pacing from a lower pacing frequency. These results might help us think about the mechanisms embodied in the aphorism ‘atrial fibrillation begets atrial fibrillation’, namely, that partially remodelled tissue could emerge in an intermediate stage of disease progression, promoting the conditions for the tissue to progress to becoming globally remodelled.

## 1 Introduction

The beating of the heart is maintained by a complex system of interacting components at varying scales, which interact in such a way that a regular rhythm emerges. In the complex, non-stationary environment that the heart finds itself, the maintenance of a regular heart rhythm demands that the constituent components of the organ at smaller scales be able to adapt their behaviour in order to maintain the necessary collective behaviour. This can lead to pathological changes, as can be seen in the phenomenon of atrial remodelling, which is associated with atrial fibrillation (AF) (Nattel and Harada, 2014). This phenomenon encompasses a series of changes in ion channels, calcium handling and structural properties that occur under long durations of high-frequency pacing, which may originate from protective mechanisms, but ultimately result in a landscape more conducive to AF (by the shortening of the action potential (AP) for example) (Nattel, 1999). AF itself creates conditions of repeated high frequency pacing near re-entrant circuits, hence the phrase ‘AF begets AF’ (Wijffels et al., 1995), which reflects the fact that AF reproduces the conditions necessary for its own maintenance through progressive remodelling.

In computational studies, one can mimic remodelling by changing the model parameters. At the tissue level setting, the atria are typically partitioned into regions (e.g., left atrium, right atrium, pulmonary veins, etc.) and the parameters are typically uniform within these regions. The assumption of a uniform parameter regime within a given region is sometimes broken, e.g., when fibrotic regions are considered (Clayton, 2018) or if a spatial gradient in the parameters is used (Ten Tusscher and Panfilov, 2003). When electrical remodelling is considered in a given region, typically the parameters within that region are altered uniformly, hence there is an implicit assumption that the said region is either globally ‘normal’ or globally remodelled^1^. Current studies therefore miss the possibility that remodelling could develop in a spatially heterogeneous fashion at a finer scale. Due to the complex structure of the tissue, one may think it plausible that such a heterogeneous emergence of remodelling would be possible. Indeed, there is some experimental evidence that ionic remodelling does develop non-uniformly in some cases, e.g., tissue from subjects with paroxysmal AF (Ramdat Misier et al., 1992) and atrial tachycardia (Fareh et al., 1998) displays intra-regional heterogeneity in refractory period durations. While certain aspects of structural remodelling (which necessarily introduces heterogeneity) have been considered in computational studies (Alonso and Bär, 2013; Vigmond et al., 2016; Clayton, 2018), there is little work in the context of spatially heterogeneous ionic remodelling, which is the topic of this paper.

Concretely, in this manuscript we will consider the effects of pacing a simulated patch of tissue that is comprised of regions in one of two states: the normal state and the remodelled state. The difference between the two states is reflected in the model parameters; in particular we use the established Grandi model of atrial AP (Grandi et al., 2011), in which the authors have specified sets of parameters for cells in sinus rhythm and cells in chronic AF, which we take as representative of the normal and remodelled states respectively. In addition to the usual ion-channel remodelling, we also assume a smaller conductivity in the remodelled regions, in accordance with alterations in gap junctions observed in remodelled myocytes (Nattel and Harada, 2014). To distribute the heterogeneity, we use the well known Ising model from statistical physics. Traditionally a model of ferromagnetism, in our case the Ising model is best understood as a random cluster model (Fortuin and Kasteleyn, 1972) (so is bond percolation, which has been used in other studies, e.g., Vigmond et al. (2016)), where the clusters correspond to remodelled regions randomly distributed in an ambient space of normal tissue. Broadly speaking, our results demonstrate three general points:

1. At sufficiently high pacing frequencies, even sparsely distributed remodelled regions can destabilise wavefront propagation, causing spatiotemporally chaotic propagation to ensue.
2. Depending on the initial condition, a long transient period of chaotic propagation may precede an eventual settling down to a periodic rhythm. This can occur when pacing frequency suddenly increases for example.
3. The dynamics depends upon how the heterogeneity is distributed, which we model by varying the coupling parameter of the Ising model.

The proposed importance of tissue heterogeneity in relation to AF can be traced back to at least the classic work of Moe and Abildskov (1959) in their elucidation of the multiple wavelets hypothesis. But since then the literature on computational models incorporating (non-structural) tissue heterogeneity has been rather sparse. Xie et al. (2001) studied the effects of a model of ventricular action potential (AP) with heterogeneous properties in the $I_{\text{K}1}$ current by randomly sampling the conductance $G_{\text{K}1}$ at each point in the finite differences lattice. They studied the effects on spiral waves, finding that the AP heterogeneity can induce spiral wave breakup. Ten Tusscher and Panfilov (2003) also use heterogeneity in ion channel conductances to study the effect on spiral wave dynamics, but their heterogeneity is based upon the assumption of a spatial gradient, rather than distributed in a random fashion. Elliott et al. (2022) incorporate cellular heterogeneity by calibrating a large set of parameters to match certain physiological biomarkers to data, and then randomly sample these to set the parameters of the individual cells. In contrast, our work only uses two different sets of parameters (normal and remodelled), which act as representatives of two polar opposites states, rather than variation within the norm. Gussak et al. (2019) study parasympathetic nerve remodelling in the left atrium, and include a computational study that is similar in flavour to this work. They show that in a model including spatially uniform parasympathetic and sympathetic signalling effects, then the two have a tendency to counteract one another allowing for stable propagation. However, introducing spatial heterogeneity in the strength of the parasympathetic effects (which is suggested by their experimental data) causes exacerbation of the pro-arrhythmic effects caused by the sympathetic signalling.

In the case of structural remodelling, e.g., fibrosis, some authors have considered a percolation type-process to represent the fibrotic regions (Cherry et al., 2007; Alonso and Bär, 2013; Vigmond et al., 2016), which relates to our approach in that bond percolation and the Ising model are both random cluster models. Fibrosis corresponds to a more advanced stage of remodelling though, and this type of approach with percolation has an effect more akin to the creation of fixed topological obstacles in the domain rather than cellular heterogeneity. Clayton (2018) also considers structural remodelling but uses a Gaussian random field to generate the remodelled regions, which are modelled as non-excitatory regions (but are still conductive via diffusion). The use of a Gaussian random field allows them to consider the effect of the scales of the heterogeneity, which is similar to this work with the Ising model.

So while spatial variability in ionic properties exist in some studies, it is typically limited to spatial gradients or the assumption of noise in specific ion channel conductances. In contrast, we consider the case in which two opposing states (remodelled and non-remodelled) coexist in randomly distributed regions.

By linking these microscopic cellular kinetics to mesoscopic structural arrangements and their combined effect on macro-scale tissue stability, this approach highlights the multiscale dynamics inherent to AP propagation in cardiac tissue. Characterizing how these structural and functional components interact to govern the transition to arrhythmia aligns with the broader principles of Network Physiology, emphasizing that tissue-level vulnerability emerges from complex interactions across distinct physiological sub-systems.

## 2 Materials and methods

To setup the general problem considered in this manuscript, we introduce the following four assumptions relating to the structure of the model of atrial tissue:

1. The tissue can be described by a 2-dimensional grid, where each region corresponds to a cell, or the average properties of a small volume of cells in close vicinity.
2. Each region can exist in one of two states, normal or remodelled, and the difference between these two states manifests itself in the ionic properties and the diffusivity at that particular site.
3. The tissue is in an equilibrium state (or a quasi-equilibrium state) so that the spatial distribution of remodelling remains constant over the time scales considered; i.e., the remodelling distribution does not evolve over the simulation.
4. Each site is correlated with neighbouring sites, so that remodelling at a site is more likely if remodelling has occurred at adjacent sites.

To resolve a concrete form for the spatial distribution of remodelling satisfying the above assumptions, we have chosen to use the well known Ising model from statistical physics.

### 2.1 The Ising model

The Ising model, which was originally formulated as a model of ferromagnetism, has found wide ranging applications across physics (Brush, 1967) and biology (Matsuda, 1981). Consider a 2-dimensional lattice L indexed by sites (i,j) , where each site is to be considered as normal or remodelled. We assign to each site (i,j) a state, denoted $r_{ij}$ , which is given by

$$r_{ij}=\left\{\begin{array}{c} 1\;\text{if remodelled}\\ -1\;\text{otherwise} \end{array}\right..$$

The Ising model describes a probability distribution over the whole configuration of sites $r\equiv {\left\{r_{ij}\right\}}_{\left(i,j\right)\in L}$ . To describe the probability distribution, we must first assign each site an energy

$$E_{ij}=-\frac{K}{2}r_{ij}\left(r_{i+1,j}+r_{i-1,j}+r_{i,j-1}+r_{i,j+1}\right)-hr_{ij},$$

where K>0 is a coupling parameter and h is an external field strength parameter. One can easily check that with the above definition, the energy at the site (i,j) is minimised when $r_{ij}$ has the same sign as h and $r_{i\pm 1,j}=r_{i,j\pm 1}=r_{ij}$ . In fact, each neighbour of $r_{ij}$ in the opposite state incurs an energy of K units. We then define the energy of the total configuration of the lattice to be the sum of energies over individual sites, $E\left(r\right)=\sum_{i,j}E_{ij}$ , and the probability of the configuration r is a Boltzmann (or Gibbs) distribution (Landau et al., 1978) based on the energy, i.e., it has the form

$$p\left(r\right)=\frac{e^{-\beta E\left(r\right)}}{Z},$$ (1)

where Z is a normalising constant and β is the inverse-temperature, which for our purposes can be assumed to be 1^2^. The characteristics of realisations of Equation 1 depend upon the coupling parameter K and the ‘external field’ parameter h , which in this scenario could be thought of as ‘remodelling propensity’^3^.

**FIGURE 1:**
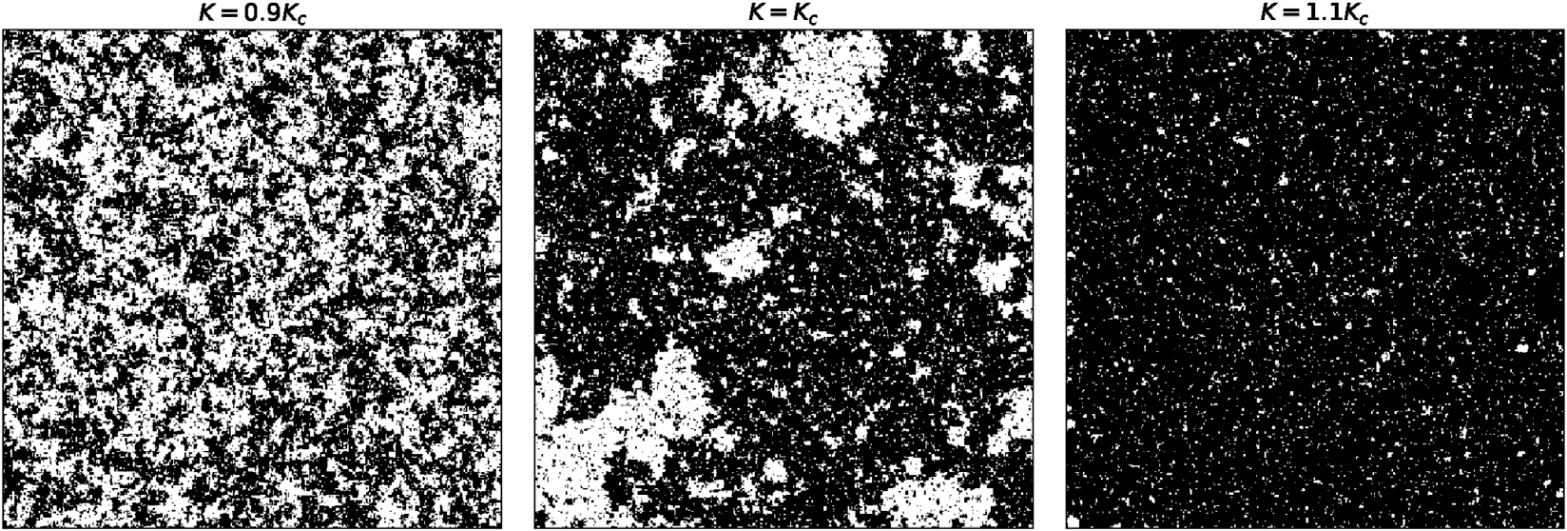
Realisations of the 2-dimensional Ising model on a 400×400 grid. White/black regions correspond to remodelled/normal tissue. The left and right plots show instances 10% below and 10% above the critical coupling respectively, and the central plot is an instance at the critical coupling.

It can be shown that in two dimensions there is a so-called critical phase of the Ising model when $K=K_{c}\equiv \log\left(1+\sqrt{2}\right)/2$ and h=0 (Gallavotti, 1999). When $K<K_{c}$ and h=0 , the coupling effect is small enough to yield significant spatial variation in typical realisations of the state, and in particular we will observe small clusters of remodelled/normal tissue (the typical size of which scale depends on how close K is to $K_{c}$ ) interwoven with one another throughout the lattice (see the left of Figure 1). If one were to zoom out far enough a realisation of the Ising model when $K<K_{c}$ would appear to resemble uncorrelated noise^4^. When $K>K_{c}$ and h=0 , the coupling is strong enough to cause a single state to engulf almost the entire lattice, with only some small clusters of the opposite state sparsely distributed across the lattice (see the right of Figure 1). If one were to zoom out in this case, the configuration would appear to be completely dominated by one particular state. When $K=K_{c}$ and h=0 , clusters of each state exist at all scales, so zooming out reveals a kind of self-similarity in the configuration, and never resembles uncorrelated noise nor the domination by a single state (see the middle of Figure 1).

In this work, we explore the effects of remodelling when it is spatially distributed according to the Ising model with h=0 and $K\in \left\{0.9K_{c},K_{c},1.1K_{c}\right\}$ . These were chosen as representative structural regimes; see Figure 1. At criticality, i.e., when $K=K_{c}$ , we are approximating the effect of large, well defined regions of remodelling that co-exist with normal tissue. When $K=1.1K_{c}$ , we are approximating the effect of remodelling emerging within a sea of normal tissue, which has not yet grown into significant remodelled regions. When $K=0.9K_{c}$ , we are approximating the effect of remodelled regions that have emerged in a more spatially uniform fashion compared to $K=K_{c}$ . So for $K=1.1K_{c}$ , we are modelling something that one may expect early on in the remodelling timeline, while the other two cases can be thought of as two different ways that remodelling could appear midway through the development of remodelled tissue.

To generate realisations of the Ising model, we use the Swendsen-Wang algorithm (Swendsen and Wang, 1987). Due to finite size effects, some realisations at criticality will include a single cluster that engulfs the bulk of the lattice, and such realisations fail to capture the full richness of the scale-free effects. To prevent such realisations from being generated, when sampling configurations at criticality we condition on the events that the proportion of tissue that is remodelled falls between 30% and 70%, and that the largest cluster of remodelled tissue is at least 5% and at most 20% of the lattice. The former condition ensures that the realisation is not almost entirely remodelled, and the latter biases the realisations towards resembling configurations where remodelled tissue has emerged from normal tissue rather than the other way around. For $K=1.1K_{c}$ , we flip the configuration (swap the labels of the normal/remodelled states) if the dominant state of the realisation is the remodelled state, ensuring that the non-remodelled state is the dominant one. The configurations when $K=0.9K_{c}$ all resemble the left of Figure 1, so no additional conditioning is required.

### 2.2 Action potential model

We use an adaptation of the Grandi model of human atrial action potential (Grandi et al., 2011), as described by Voigt et al. (2013). To model the normal state we use the typical parameters given in Grandi et al. (2011), and to model the remodelled state we use the chronic AF parameters (cAF) given in the same work. Compared to the original Grandi model, the adaptation uses the Courtemanche model (Courtemanche et al., 1998) variant of $I_{\text{Na}}$ , which has faster recovery variables than the Grandi formulation, allowing it to respond to faster pacing without propagation failure occurring.

We do not fix the sodium concentrations^5^ in the model but do fix the intracellular potassium concentration, which slowly drifts towards 0 if it is not fixed. The significance of the sodium concentration variables is that they are slowly evolving, approaching equilibrium over hundreds of beats (while the other variables typically adjust over only a few beats), and have a strong effect on the action potential duration (APD). The sodium concentration tends to increase upon faster pacing, which causes the APD to shorten and therefore allows for the faster pacing to be maintained. Abrupt increases in pacing frequency can therefore result in a long period of non-stationary behaviour as the sodium concentration adapts. See the Supplementary Material for a more detailed explanation of the dynamics of the sodium concentration in this model, and see Figure 5C in Voigt et al. (2013) for the APD rate dependence curves for both normal and remodelled parameters. At a given pacing period T , we numerically find the limit cycle for the single cell model for both sets of parameters, and set the state of the normal/remodelled regions accordingly. To find the limit cycle, we use Newton’s method on the Poincaré map of the single cell model for the two sets of parameters, which is explained in more detail in the Supplementary Material.

For normal tissue, we have assumed an effective diffusivity of $D_{\text{normal}}=0.1$ ${\text{mm}}^{2}$ /ms and an anisotropy ratio of 1. In remodelled tissue, we have assumed that some level of structural remodelling and/or gap junction downregulation has occurred and set $D_{\text{remodelled}}=0.05$ ${\text{mm}}^{2}$ /ms with an anisotropy ratio of 2, which give a similar reduction in diffusivity/increase in anisotropy ratio in Varela et al. (2016). Note that real tissue is not isotropic, however some studies have found that the effective anisotropy ratio is close to 1 in healthy atrial tissue (Houben et al., 2004). Moreover, patients with long term persistent AF have been measured to exhibit a greater degree of anisotropy than those with acute AF (van Schie et al., 2024), suggesting that a larger anisotropy ratio in the remodelled tissue serves as a reasonable phenomenological baseline. With entirely non-remodelled tissue, these parameters yield a conduction velocity of approximately 0.5 m/s (pacing at T=1000 ms), and when heterogeneity is sampled from the critical Ising model $\left(K=K_{c}\right)$ , the conduction velocity only slightly decreases to approximately 0.45 m/s. These conduction velocities seem reasonable in the current context given that they are within a physiological range (they are on the lower end of what one might observe in induced AF and within the normal range for persistent AF (van Schie et al., 2024)). Details of the numerical methods used for simulation are outlined in the Supplementary Material.

### 2.3 Simulation details and metrics

For all experiments we use a 400×400 grid with a spatial step of Δx=0.15 mm, and a time step of $\Delta t=5\times 10^{-3}$ ms. We used the monodomain model to model propagation, which was implemented numerically using a finite volume method (see Supplementary Material for details). To stimulate wave propagation, a stimulus current was periodically applied at the bottom edge of the grid so that wavefronts travel upwards (in the absence of condition blocks at least). Unless otherwise stated, for each experiment we run the simulation for 2,000 beats. We use 10 instances of heterogeneity when considering $K=K_{c}$ for each set of pacing conditions (with the exception of the one reported in Section 3.3), as the critical Ising model displays significant variability at the macroscopic level, as shown in Figure 1. For $K\neq K_{c}$ , we only use one realisation since the distribution of remodelled regions is essentially isotropic away from the critical point. We record the action potential $V_{m}$ and intracellular sodium concentration ${\left[Na\right]}_{i}$ at the start of each beat and halfway between two beats, i.e., if the pacing period is T ms, we collect the two quantitites every T/2 ms. We also record the spatial mean of $V_{m}$ every ms, which works well as a one dimensional summary of the global behaviour of propagation.

At low pacing frequencies, we get a stable, periodic propagation regime, while at very high pacing frequencies, we get a period doubling effect^6^, as the stimulus arrives before the tissue has fully repolarised. It can be difficult to assess periodic behaviour of the system visually due to it is high dimensionality, so we must use an appropriate metric to assess this. Note that for a system in discrete time, say $y_{t}$ , k− periodic behaviour implies that $|y_{t+k}-y_{t}|=0$ for all t , and if we perturb the state slightly (and the k− periodic behaviour is stable), we would expect that $|y_{t+k}-y_{t}|∼e^{-\lambda t}$ for some λ>0 . We can construct a discrete time system from the data collected by taking $V_{m}\left(t,x\right)$ at the time of each stimulus, i.e., $y_{n}=V_{m}\left(nT,x\right)$ . We then use the metric given by:

$$d_{n}^{\left(k\right)}\equiv \frac{1}{k}\overset{k-1}{\underset{i=0}{\sum }}\frac{1}{\left|D\right|}\int_{D}\left|V_{m}\left(\left(k\left(n+1\right)+i\right)T,x\right)-V_{m}\left(\left(kn+i\right)T,x\right)\right|dx,$$ (2)

where $d_{n}^{\left(k\right)}$ denotes the distance metric for the $n^{\text{th}}$ cycle assuming period k , and |D| denotes the volume of the domain. Note that for k=1 , this reduces to the spatial average of the difference between $V_{m}$ at successive beats, which will decay to 0 exponentially quickly if a stable periodic limit cycle is approached. For k=2 there are two beats in a cycle, so the measure takes the distance between the first beats of two successive cycles and the distance between the second beats of the two successive cycles and averages them. For larger values of k , the interpretation is similar: there are k beats within a cycle, so we average over the distances obtained by choosing each of the k possible beats as representative of the cycle. For experiments with $K=K_{c}$ , the values of $d_{n}^{\left(k\right)}$ that we report correspond to the average over the 10 instances of heterogeneity unless otherwise stated.

Further details with regards to numerical methods are given in the Supplementary Material.

## 3 Results

### 3.1 General behaviour

**FIGURE 2:**
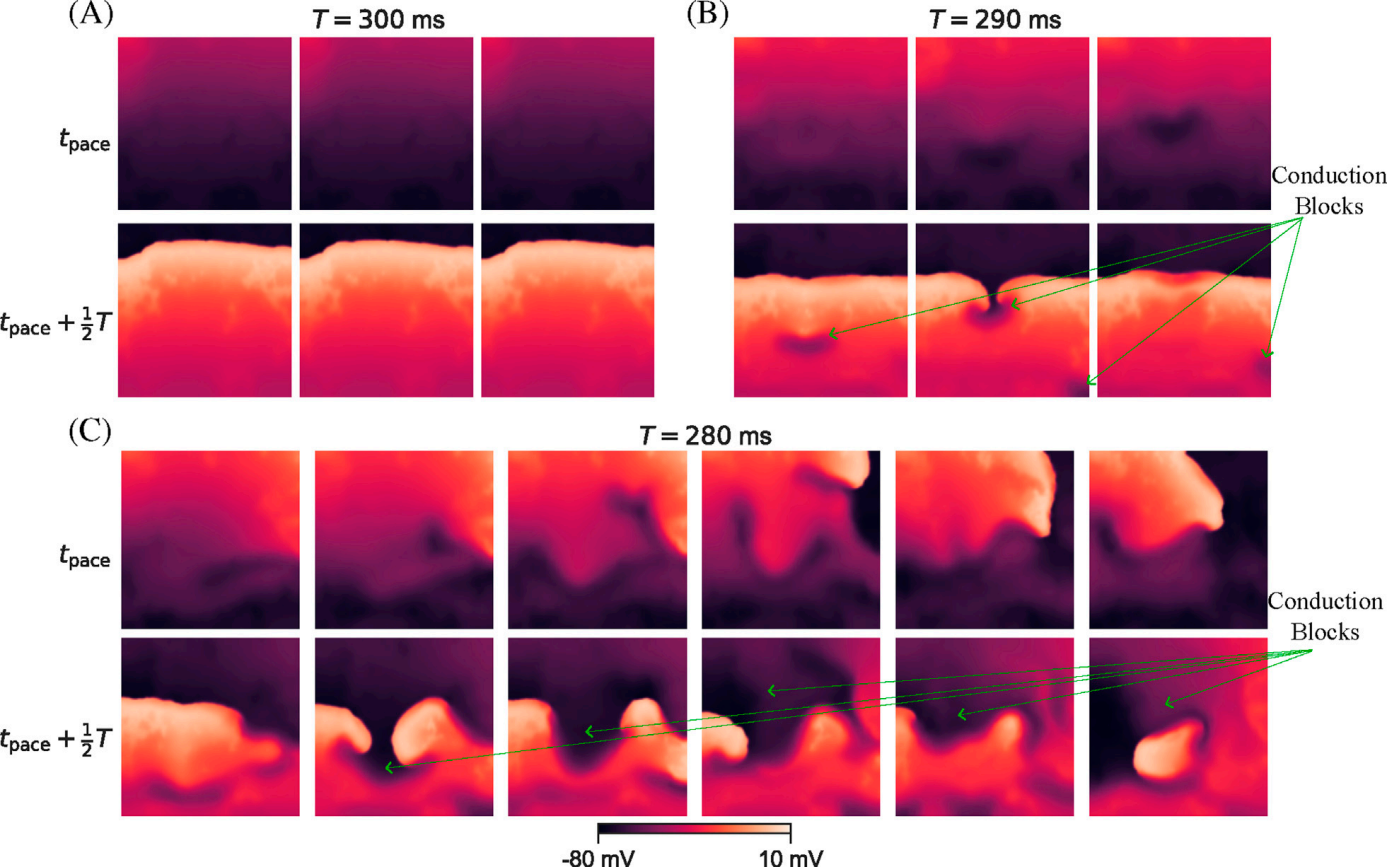
Examples of AP propagation with $K=K_{c}$ for different values of T . **(A)** T=300 ms for beat numbers 998–1,000 (from left to right), where the top image in each column shows the AP at the time of stimulus $t_{\text{pace}}$ and the bottom shows the state half way between stimuli. **(B)** T=290 ms for beats 998–1,000. Visible conduction blocks have also been labelled. **(C)** T=280 ms for beats 995–1,000. Significant conduction blocks have been labelled in this case.

First consider Figure 2, which displays example snapshots of AP for a particular instance of $K=K_{c}$ and cycle lengths T=300 ms, T=290 ms and T=280 ms. This broadly displays the types of qualitative behaviour that we see throughout the experiments, namely, regular periodic propagation (for T=300 ms, panel A), irregular but still relatively laminar propagation (for T=290 ms, panel B), and irregular, turbulent behaviour ( T=280 ms, panel C). Also indicated are some regions of conduction block that emerge in the T=290 ms experiment shown. These regions, which are not fixed in space, regenerate themselves by destabilizing the shape of the wavefront, leading to unpredictability in the AP at the local level (but globally the behaviour is quite coherent). In Figure 2B ( T=290 ms), we see that when the wavefront encounters the block a break occurs, but it is sufficiently small as to allow the wavefront to subsequently re-merge into a single coherent front. For the T=280 ms experiment shown (Figure 2C), it is clear that the conduction blocks occupy much larger regions with a less regular shape, thus creating pathways for turbulent re-entrant circuits to emerge.

### 3.2 Effect of pacing period and coupling

**FIGURE 3:**
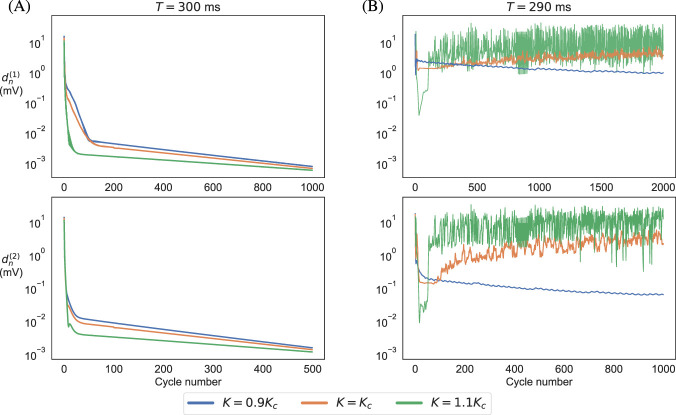
Difference metrics $d_{n}^{\left(1\right)}$ and $d_{n}^{\left(2\right)}$ for T=300 ms and 290 m (see Equation 2). **(A)** T=300 ms. It is evident from the top panel (showing $d_{n}^{\left(1\right)}$ ) that the difference between $V_{m}$ over successive beats decays exponentially for all experiments, indicating periodicity. The bottom panel shows the same for $d_{n}^{\left(2\right)}$ . Note that the sharp drops near the beginning reflect transient behaviour due to the initial conditions. **(B)** T=290 ms. In this case neither $K=K_{c}$ nor $K=1.1K_{c}$ displays periodic behaviour, except for a short transient period of period 2 like behaviour (indicated by small values of $d_{n}^{\left(2\right)}$ in the bottom panel), but $K=0.9K_{c}$ does exhibit period 2 behaviour, although the plateau in the decay (indicated by the decreasing slope in the bottom panel) suggests that this behaviour may not be stable.

All instances of experiments with T=300 ms show the emergence of a period 1 limit cycle. This is made evident in the upper plot in Figure 3A which shows the time series of $d_{n}^{\left(1\right)}$ values defined in Equation 2. Note that because periodic behaviour is obtained for all experiments with T=300 ms, we have only run the simulation for 1,000 beats rather than 2,000. An example of the propagation can be seen in Figure 2A. We can see that after an initial transient period (corresponding to the tissue adapting to pacing) the lag 1 distances decay exponentially, which is what we would expect for a system approaching a stable attractor. Moreover, the lag 1 distance decays sharply from the outset, showing that there is a lack of chaotic transient behaviour.

**FIGURE 4:**
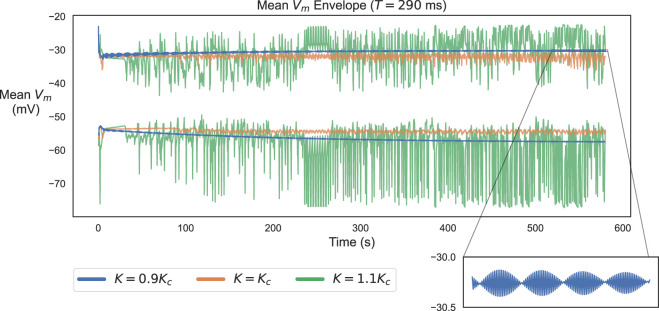
Envelope of $V_{m}$ spatial mean for T=290 ms experiments. The plot shows the behaviour for different values of K , where we are only showing one instance of $K=K_{c}$ . The inset displays the upper envelop of the $K=0.9K_{c}$ experiment over the last 200 beats, and shows that there is small scale oscillatory behaviour present.

The behaviour when T=290 ms can be inferred from Figure 3B, which shows the lag 1 and lag 2 distances $d_{n}^{\left(1\right)}$ and $d_{n}^{\left(2\right)}$ , and Figure 4, which shows the envelope of the spatial average of $V_{m}$ . In all cases, the lag 1 distances (Figure 3B) are on the order of 1–10 mV (except for a brief period near the beginning of the simulation when $K=1.1K_{c}$ ). On the other hand, we can see that for all values of K , there is a period where the lag 2 distances $d_{n}^{\left(2\right)}$ are small (on the order of 0.1 mV), indicating period 2 like behaviour in all experiments. Qualitatively, this corresponds to laminar wavefronts with a slightly different shape on alternating beats. When $K=0.9K_{c}$ , one can see from Figure 3B that this behaviour persists for the entire 2000 beats, however the characteristics of the dynamics are not the same as the exponential decay seen in $d_{n}^{\left(1\right)}$ and $d_{n}^{\left(2\right)}$ when T=300 ms: a slowing in the rate of decay is observed along with small oscillations. The same type of oscillatory behaviour is observed when $K=K_{c}$ over the first 250 beats, before irregular behaviour sets in. These small scale irregular oscillations are manifested in the spatial average of $V_{m}$ as well, as shown in Figure 4. This suggests that the apparent period 2 behaviour for $K=0.9K_{c}$ may be a transient phenomenon, which would not be sustained if we were to run the simulation for a longer period of time. After the transient period 2 behaviour ceases in the $K=K_{c}$ example, we observe an intermediate state between laminarity and turbulence, characterised by small wave breaks but still relatively steady propagation as in Figure 2B, while the dynamics of propagation for $K=1.1K_{c}$ consists of larger wave breaks and irregular patterns of propagation. One can infer that there is a qualitative difference in propagation between $K=1.1K_{c}$ and $K=K_{c}$ by the fact that there is roughly an order of magnitude difference between the respective lag 1 and 2 distances in Figure 3B, indicating significantly greater spatial variability in the AP at each beat for $K=1.1K_{c}$ . This is also clearly manifested in Figure 4, which shows large, aperiodic fluctuations in the amplitude of the spatial mean of $V_{m}$ for $K=1.1K_{c}$ , but only small fluctuations for $K=K_{c}$ . We have included additional AP heatmaps for $K=K_{c}$ and $K=1.1K_{c}$ in the Supplementary Figures to visually demonstrate the difference between the propagation behaviours.

**FIGURE 5:**
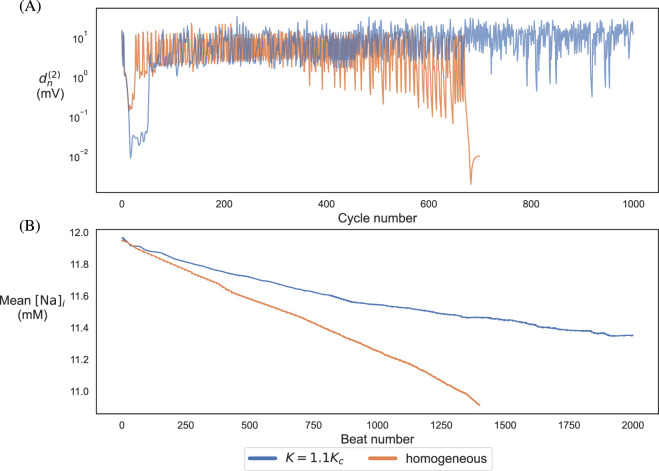
**(A)** Values of $d_{n}^{\left(2\right)}$ for homogeneous model (with non-remodelled parameters) compared to the experiment with $K=1.1K_{c}$ , both paced at T=290 ms. It can be inferred from this that in the homogeneous model, uniform propagation failure occurs regularly before transitioning to periodic propagation failure at around cycle 700. The intermittent drops for $K=1.1K_{c}$ are much more sporadic in comparison. **(B)** Average sodium concentration for homogeneous model and $K=1.1K_{c}$ (in the non-remodelled regions only). In the homogeneous model, the decline is approximately linear until propagation failure finally becomes periodic in which case it accelerates, while the decline is much slower for $K=1.1K_{c}$ , and appears to plateau towards the end. This suggests that the turbulent behaviour observed for $K=1.1K_{c}$ may be stable.

As a point of comparison, a completely homogeneous model with non-remodelled parameters transitions to period 2 behaviour where a uniform conduction block prevents propagation every other beat. Initially, all beats propagate due to an initially high sodium concentration, which slowly drops across the tissue causing a slow lengthening of the APD. This causes the tissue to exhibit behaviour in which propagation fails in a quasi-periodic manner, which drives further depletion of sodium (via failure to activate $I_{Na}$ ) and eventual uniform conduction block; see Figure 5. From Figure 5A, it is evident that episodes of propagation failure occur intermittently for $K=1.1K_{c}$ (shown by the drops in $d_{n}^{\left(2\right)}$ ) from approximately cycle 600 onward, but they are far less regular in the homogeneous model before the transition to period 2 behaviour. One can also see a difference in the dynamics of the average intracellular sodium concentration in Figure 5B, with the homogeneous model experiencing a linear decline up to the transition (after which the decline accelerates), while for $K=1.1K_{c}$ , the sodium concentration is slower to decline and appears to plateau, suggesting that the irregular behaviour may persist indefinitely.

**FIGURE 6:**
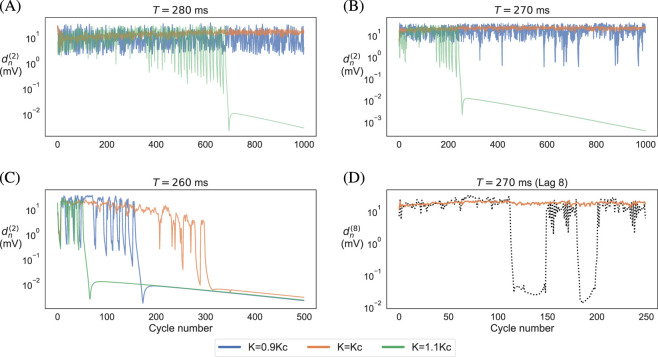
$d_{n}^{\left(2\right)}$ shown for **(A)** T=280 ms. **(B)** T=270 ms, and **(C)** T=260 ms. Evidently, for T=270 ms and 280 m, $K=0.9K_{c}$ and $K=1.1K_{c}$ exhibit aperiodic, unpredictable behaviour (Note that for $K=K_{c}$ , the average over 10 instances of heterogeneity is shown, which dampens the fluctuations compared to $K=0.9K_{c}$ ), and for T=260 ms they both fall into period 2 behaviour. $K=1.1K_{c}$ always falls into period 2 behaviour, however it displays a long transient of aperiodic behaviour for T=280 ms, which shortens for T=270 ms (and still more for T=260 ms). **(D)** $d_{n}^{\left(8\right)}$ for $K=K_{c}$ and T=280 ms. The solid orange line shows the average of $d_{n}^{\left(8\right)}$ over the 10 instances of heterogeneity as a reference, and the black dashed line shows a single instance that exhibits transient episodes of period 8 behaviour.

For T=280 ms, we observe turbulent, aperiodic behaviour in all cases, with the exception of when $K=1.1K_{c}$ , where we observe a transient period of turbulent behaviour followed by a doubling of the period, corresponding to the stimulus failing to generate a propagating wavefront every other beat. This can be inferred from Figure 6A, where it is evident that the lag 2 distances decay exponentially for $K=1.1K_{c}$ (but remain large and unpredictable when $K<1.1K_{c}$ ). We see similar behaviour when T=270 ms (shown in Figure 6B), only with a shorter transient period of turbulent behaviour when $K=1.1K_{c}$ . When T=260 ms, all experiments experienced period doubling within 1,000 beats (Figure 6C). Once period doubling sets in, the effective pacing period becomes 2T , e.g., 520 ms for T=260 ms, and as we have seen, stable propagation can be achieved at periods as low as T=300 ms, so the alternation between successful and failed propagation ultimately induces stable behaviour. Figure 6D shows the lag 8 distance $d_{n}^{\left(8\right)}$ for one particular experiment with $K=K_{c}$ and T=270 ms (compared to the average over the 10 instances when $K=K_{c}$ ). We show this because the experiment in question demonstrated an interesting effect, where an apparent limit cycle with period 8 was approached on two separate occasions, and not only this but it appeared to be relatively stable. However, it ultimately proved to be unstable as the sudden return to large values of $d_{n}^{\left(8\right)}$ in Figure 6D shows. This demonstrates a type of intermittent behaviour in the model; a visualization of the period 8 behaviour is shown in the Supplementary Figures.

**FIGURE 7:**
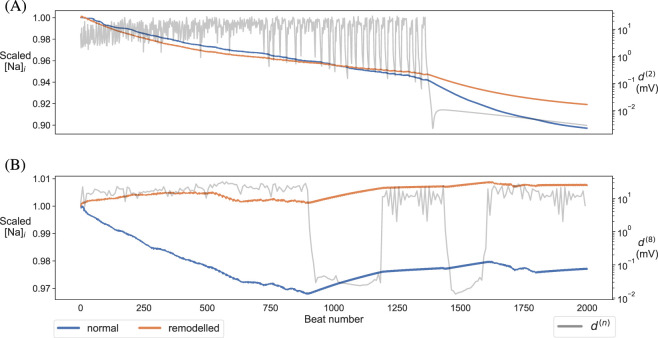
Average ${\left[Na\right]}_{i}$ in proportion of its initial value in remodelled and non-remodelled regions. **(A)** T=280 ms and $K=1.1K_{c}$ , along with the value of $d_{n}^{\left(2\right)}$ (with the x-axis scaled to match the beat number) shown on the right axis as a reference. **(B)** An instance of $K=K_{c}$ and T=270 ms with transient period 8 behaviour (see Figure 6D). The right axis shows $d_{n}^{\left(8\right)}$ as a reference.

Notice that for the $K=1.1K_{c}$ experiments in Figure 6A ( T=280 ms) and Figure 6B ( T=270 ms), before approaching the period 2 limit cycle, the system has several periods where the lag 2 distances drop significantly, but subsequently rise to large values rather quickly. These correspond to short bouts of periodic behaviour that quickly lose stability, and suggest a slow time scale in the system that controls the stability of the emergent limit cycle. We visualise this in Figure 7 by showing the evolution of the slowly evolving average ${\left[Na\right]}_{i}$ (partitioned into remodelled and non-remodelled areas). Figure 7A shows that the values of $d_{n}^{\left(2\right)}$ for T=280 ms appear to be correlated with the declining average ${\left[Na\right]}_{i}$ , and when the period doubling sets in, the rate of decay of ${\left[Na\right]}_{i}$ undergoes a sudden increase (until it eventually plateaus towards an equilibrium value). A similar analysis of the case shown in Figure 6D ( T=270 ms, $K=K_{c}$ and near period 8 behaviour) is shown in Figure 7B. In this case, ${\left[Na\right]}_{i}$ in the remodelled areas remains roughly steady over the first 900 beats or so, whilst it drops in the non-remodelled areas. But when the near periodic behaviour emerges, a sufficiently stable propagation regime is sustained as to cause average ${\left[Na\right]}_{i}$ to rise globally, which presumably lays the foundations for this cycle to become unstable once again by shortening the APD (and hence the length of the wave back) allowing propagation to break through in regions where it was previously blocked in the period 8 cycle.

### 3.3 Effect of initial condition

Here we consider the effect of an abrupt change in pacing period on the dynamics. In the experiments previously discussed, we have assumed that the tissue has already approximately adjusted to the pacing conditions by exploiting the limit cycle behaviour of the single cell model. In reality however, we might expect increases in pacing frequency to occur suddenly due to some external trigger, in which case the tissue would have to go through a transient period where it adapts to the new pacing frequency. We approximate this behaviour by initializing the state based on the single cell model paced every 600 m, and commence pacing the tissue with T=300 ms.

**FIGURE 8:**
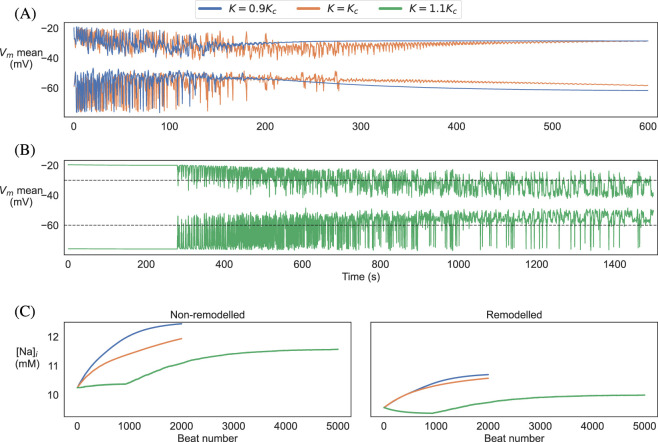
T=300 ms with altered initial condition. **(A)** Envelope of spatial mean of $V_{m}$ observable for $K=0.9K_{c}$ and $K=K_{c}$ , which shows an initial period of chaotic behaviour that eventually settles down to periodic behaviour. **(B)** Envelope of spatial mean of $V_{m}$ for $K=1.1K_{c}$ , which shows an initial period of stable periodic behaviour (corresponding to propagation failure) followed by persistent chaotic behaviour. **(C)** Average ${\left[Na\right]}_{i}$ for non-remodelled regions (left) and remodelled regions (right).

Figure 8 displays the essence of the behaviour for the three couplings considered. Figures 8A,B show the envelope of the spatial average of $V_{m}$ over the entire simulations, the former showing $K=0.9K_{c}$ and $K=K_{c}$ , and the latter showing $K=1.1K_{c}$ . We have separated the latter instance as we have run this for a total of 5,000 beats as opposed to 2,000 beats. The reason for the extension of run time will become evident below. We also provide the plots of the distances $d_{n}^{\left(1\right)}$ and $d_{n}^{\left(2\right)}$ for each of these experiments in the Supplementary Figures. For $K=0.9K_{c}$ and $K=K_{c}$ , we can see that turbulent behaviour commences from the outset (evidenced by the large fluctuations in amplitude displayed in Figure 8A), but gradually subsides and is replaced by the periodic rhythm observed in Figure 2A. This shows that transient episodes of chaotic behaviour are possible even when a stable limit cycle exists, and can come about due to a sudden bout of fast pacing. The duration of the turbulent period is longer for $K=K_{c}$ than it is for $K=0.9K_{c}$ , and moreover the adaptation to periodic behaviour is more gradual.

For $K=1.1K_{c}$ the behaviour is rather different. In the first 300 s of the simulation there is propagation failure every other beat (evidenced by the constant envelope with large amplitude seen in Figure 8B). After the 300 s mark however, there is a sudden transition to chaotic behaviour, which then persists for 1,200 s (4,000 beats). This suggests the opposite of what we described above for $K<1.1K_{c}$ : initially the system is able to maintain a periodic rhythm at the original pacing period ( T=600 ms) and does not allow for propagation every second beat. But after repeatedly pacing the tissue for a sufficiently long duration, portions of the wavefront begin to break through the blocked region and turbulent propagation ensues, and evidently does not stop.

Figure 8C shows the dynamics of the average sodium concentration in the remodelled and non-remodelled areas. At a pacing period of T=600 ms, the equilibrium sodium concentration is significantly lower than when T=300 ms, particularly in the non-remodelled regions, which induces longer wave backs, explaining the initial turbulence for $K=0.9K_{c}$ and $K=K_{c}$ , and the initial propagation failure for $K=1.1K_{c}$ . We can also see that the rate of increase of sodium in the non-remodelled regions is significantly greater for $K=0.9K_{c}$ than it is for $K=K_{c}$ (though this difference is less pronounced for the remodelled areas), and appears to be approaching a plateau by beat number 2000, while it is still steadily increasing for $K=K_{c}$ . This would explain why the $K=K_{c}$ example takes much longer to settle down to the limit cycle compared to $K=0.9K_{c}$ . Presumably, the more equidistributed remodelling when $K=0.9K_{c}$ has the effect of shortening the APD in non-remodelled areas via diffusive effects, allowing depolarization to occur more uniformly over time (and hence increasing the average sodium concentration), whereas for $K=K_{c}$ , this would tend to be localised around large remodelled clusters. For $K=1.1K_{c}$ , we see a very slow increase in the sodium concentration over the first 1,000 beats in the non-remodelled regions, and a decrease in the remodelled regions^7^, which experience a sudden increase in growth rate when turbulence sets in after 1,000 beats. This is not surprising: the slow increase in sodium concentration in the non-remodelled regions over the first 1,000 beats reflects an increase in sodium concentration in the immediate proximity of the edge where pacing is applied (but the rest of the domain experiences little movement in sodium concentration due to propagation failure), shortening the APD in this region and the surrounding regions due to diffusive effects. Evidently, the sodium concentration across this region increases up to a threshold upon which the APD in this region becomes just short enough to allow wavefronts to break through certain regions that were previously blocked by refractory tissue. The reason we extended the $K=1.1K_{c}$ experiment was in anticipation of an eventual transition to periodicity like the other two examples. However, the sodium concentration appears to have approached an equilibrium by beat number 5,000, suggesting that such a transition may not occur at all. Moreover, in the aforementioned region along the paced edge, the sodium concentration actually begins to slowly decrease after approximately 3,500 beats, which can be seen in the Supplementary Figures. This suggests that periodic behaviour may not be achieved at all in this example, and that the turbulent behaviour may be sustained indefinitely.

## 4 Discussion

In this work we investigated how the spatial organisation of electrical remodelling influences wave propagation in atrial tissue. While it is well established that heterogeneity can promote conduction block and arrhythmogenesis, remodelling is often represented as a property of entire regions rather than as a spatially emergent process occurring at mesoscopic scales. By considering remodelled tissue as clusters embedded within a sea of non-remodelled tissue, we were able to examine how the degree of spatial organisation influences the resulting dynamics. A key observation is that the spatial organisation of remodelling can have an effect on the propagation dynamics, and in particular it often manifests through the length of transient episodes of turbulent behaviour (as is made evident in, e.g., Figures 6, 8). Our analysis also revealed the importance of ultra-slow sodium-mediated adaptation (the dynamics of which are also dependent on the distribution of remodelling) in maintaining the different dynamical regimes observed. These results suggest that the spatial structure of remodelling may be as important as its overall extent when determining tissue-level dynamics.

### 4.1 The effects of diffusivity

We have seen that if carefully initialised, all experiments exhibited regular periodic propagation with pacing period T=300 ms, but developed more complex behaviour at all the faster pacing frequencies. To explain why these different behaviours occurred, we first note some general dynamical features that we would expect to see based on the structure of the model. One thing that must be considered when comparing the results is that we halved the diffusivity in the remodelled areas, so the effective diffusivity for $K=1.1K_{c}$ is larger than for the other two cases, which has implications for the behaviour observed in the corresponding experiments. If we were to consider the model in the case of a (homogeneous) one-dimensional cable, then simple scaling relations imply that varying the diffusivity D will cause the conduction velocity and wavelength to scale like $\sqrt{D}$ . In the case of an sufficiently long cable, if periodic pacing results in stable behaviour, then the induced wave travels fast enough such that the refractory wave back drops to resting potential by the time the next stimulus arrives, allowing another wave to propagate. Increasing D increases the wavelength and hence the mass of refractory tissue, but the increased wave speed will compensate for this and the tissue will still drop to resting potential at the next stimulus. However, when D is large enough such that the wavelength becomes comparable with the domain size, then boundary effects become noticeable, and in particular, once the wavefront approaches the boundary, the forward movement of the wave stops and hence the rate at which the wave back drops to resting potential decreases, resulting in an increased likelihood of propagation failure. Hence for $K=1.1K_{c}$ (where only approximately 5% of the domain is remodelled), it is not surprising that the system experiences regular propagation failure for T≤280 ms, whereas for the other two values of K (where 30%–50% of the domain is remodelled), it only occurs at T=260 ms.

In addition to the effects of the altered diffusivity, the altered parameters reflecting ionic remodelling exert an effect on tissue level refractoriness via the cellular level effects on APD. In the single cell model, remodelling causes the APD to become relatively insensitive to changing pacing frequencies, and in particular it is much shorter than the APD of the non-remodelled model at low pacing-frequencies (Voigt et al., 2013). This then gives us an indication as to why in Section 3.3, $K=1.1K_{c}$ undergoes a long period of propagation failure, while the other two values of K do not: Since we initialise according to T=600 ms, the non-remodelled tissue exhibits a significantly longer APD than it would if we initialised according to T=300 ms, however for the remodelled tissue the increase in APD is much less pronounced, which allows propagation to occur in remodelled regions without a long period of adaptation. As T approaches 300 ms, the two APDs (measured as the APD90) become quite close (Voigt et al., 2013). However with the remodelled parameters, the AP shape is much more ‘triangular’ than in the non-remodelled case, i.e., in the remodelled cells the AP drops to resting potential in a linearly fashion, while the non-remodelled cells exhibit a slower plateau towards resting potential from the point it reaches its APD90 (see Supplementary Material to see how the shapes differ). If T=290 ms, for example, with remodelled parameters the AP is about 5 mV lower than for the non-remodelled parameters at the time of stimulation. This is significant, because when diffusion is involved, the effect will be that on average, the AP in non-remodelled regions is lowered slightly (and conversely for remodelled tissue), accelerating repolarization. The distribution of remodelled tissue is significant in this sense too: the diffusive effects of a small cluster of remodelled tissue will be dominated by the influence of a large surrounding region of non-remodelled tissue, however a large cluster of remodelled tissue allows the characteristics of the remodelled AP to persist in this region, which can then exert an effect on non-remodelled tissue near the boundary of the cluster. It is in this sense that we may be able to explain the differences in behaviour between $K=K_{c}$ and $K=0.9K_{c}$ : the homogeneous distribution of remodelling for $K=0.9K_{c}$ creates a stronger synchronization effect between regions (although small scale spatial fluctuations still exist), while when $K=K_{c}$ , we get larger, more well defined regions with differing AP characteristics.

It is clear from the above discussion that some of the particularities of the behaviour observed may be peculiar to the Grandi model and/or the value of the diffusivity used. It would be conceptually straightforward to repeat the experiments performed here with a different ionic model such as the Courtemanche model (Courtemanche et al., 1998), or to test the results of varying the diffusivity/domain size. The same can be said of the anisotropy, which we have not investigated the effect of in any detail here. We leave a systematic exploration of the effects of these factors as future work.

### 4.2 The effects of increasing pacing frequency

When T=290 ms we saw that experiments with $K=K_{c}$ and $K=1.1K_{c}$ differed qualitatively in how they deviated from regular periodic behaviour, with the latter displaying turbulent propagation and the former displaying chaotic behaviour, but not what we would think of as turbulence (e.g., large wave breaks, re-entrant circuits etc.,). For $K=K_{c}$ , we saw what mediates this behaviour in Figure 2B, namely, the development of small, irregular conduction blocks corresponding to regions of incomplete repolarization. When $K=1.1K_{c}$ , the emergence of aperiodic behaviour happens to occur in a similar fashion, however the faster wave speed (see Section 4.1) causes newly formed fronts to encounter the blocks faster, causing them to grow larger and rather quickly provoke more significant wave breaks than what is seen in Figure 2B. What is interesting is that the remodelled regions appear to have a dual effect on the dynamics: on the one hand sparsely distributed remodelled regions are sufficient to destabilise the dynamics enough to promote an environment of constantly reproducing conduction blocks, driving turbulent propagation. On the other hand, when remodelled regions are sufficiently large, the opposite effect is produced, i.e., the remodelled regions create a stabilizing effect by shortening the average wavelength and conduction velocity, which helps to control the size of the conduction blocks by reducing the mass of refractory tissue (see the discussion in Section 4.1).

A possible dynamical systems interpretation for the turbulent behaviour and chaotic non-turbulent behaviour comes from the notion of an interior crisis (Grebogi et al., 1983). This occurs when an unstable limit cycle collides with a chaotic attractor, causing a sudden widening of the attractor. We suggest this because on the one hand, if we use a homogeneous, non-remodelled model with T=290 ms, there is a stable limit cycle in which propagation fails every other beat after a transient period of quasi-periodic propagation failure^8^, and we expect that a slight perturbation in the parameters^9^ only slightly perturbs the limit cycle, however it need not be stable after the perturbation. On the other hand, when $K=K_{c}$ we see unpredictable but globally coherent behaviour, which could plausibly correspond to motion along a low dimensional chaotic attractor. One could then imagine that interpolating between the two regimes would lead the unstable limit cycle and chaotic attractor to collide, resulting in dynamics that exhibit large fluctuations between beats. The result would then be intermittent episodes of motion that follows the unstable limit cycle, or in the terms of the system at hand, intermittent episodes of propagation failure. Indeed, this type of effect can be observed in some of the T=290 ms experiments with $K=K_{c}$ (though not all of them, it appears to be dependent on the Ising model realisation), where the dominant dynamics are like those in Figure 2B, but with occasional episodes of re-entrant like behaviour that causes the subsequent beat to be blocked. We show an example of this in the Supplementary Figures.

For T=280 ms we see that $K=K_{c}$ (and $K=0.9K_{c}$ ) both undergo a transition to turbulent behaviour, so the aforementioned stabilizing effect of remodelling only manifests itself over a small window of pacing periods. The mechanism for this is a straightforward extension of what we described above, namely, that wave fronts encounter conduction blocks faster, causing a reverberation effect to take place and eventually spark turbulent behaviour. The only difference in this case is that a shorter interval between stimuli provokes the behaviour, rather than an increased conduction velocity. When $K=1.1K_{c}$ , we see a long transient period of turbulent behaviour, which proves to be unstable and eventually gives way to period doubling, where the stimulus fails to provoke a wavefront every other beat. When T=270 ms, we largely see the same behaviour as when T=280 ms, the main difference of note being that when $K=1.1K_{c}$ , the transient period of turbulent behaviour is shorter. And finally when T=260 ms, all experiments undergo a transition to propagation failure, showing that in all cases there is a threshold pacing that cannot be maintained. One could interpret this as a kind of ‘protective’ effect, since the threshold (in terms of T ) for repeated propagation failure is higher when the proportion of remodelled tissue is lower, which one would logically expect since remodelled tissue is thought to stabilise high frequency re-entrant circuits (Nattel et al., 2008). Nevertheless, the transient periods for sparse remodelling $\left(K=1.1K_{c}\right)$ can last for long durations (For T=280 ms it lasts for approximately 1,400 beats, for example), so one could imagine that a growing fraction of remodelled tissue results in the lowering of this pacing threshold, and the prolongation of the average length of the transient until the turbulent behaviour becomes stable. In the experimental work of Wijffels et al. (1995), there is a similar transition in the duration of AF episodes relative to the amount of time that AF was induced beforehand using a pacemaker (which one would expect to be correlated with the degree of remodelling present), where the duration of triggered AF episodes rise sharply from a few seconds up to weeks as the duration connected to the AF pacemaker rises.

We note that the aforementioned notion of an interior crisis could plausibly apply to explain the behaviour of the transition to turbulent behaviour for $K<1.1K_{c}$ as well, see, for example, the intermittent drops in $d_{n}^{\left(2\right)}$ for T=270 ms, $K=0.9K_{c}$ in Figure 6B. However, we want to note that the exceptional case that we described when period 8 behaviour emerges intermittently ( T=270 ms, $K=K_{c}$ ) might be more resemblant of Pommeau-Manneville intermittency (Pomeau and Manneville, 1980), where trajectories slowly diverge from an unstable limit cycle, but ultimately return to it is vicinity after a chaotic passage^10^. As for the transient episodes of turbulent behaviour when $K=1.1K_{c}$ , inspection of the lengths of the transients suggests that a so-called boundary crisis (Grebogi et al., 1983) could be a plausible explanation. When a boundary crisis occurs, a chaotic attractor is destroyed upon collision with the basin of attraction of a stable limit cycle, however there are residual effect on the dynamics in the sense that trajectories can become ‘tangled up’ for a lengthy period near the chaotic set. Moreover, if the boundary crisis occurs at some critical parameter $T_{c}$ , then the average length of the transient chaotic episode, say τ , usually follows a power law: $\tau ∼|T-T_{c}{|}^{-\gamma }$ for some exponent γ (Grebogi et al., 1987). This would explain why the duration of the transient episode is significantly shorter for T=270 ms than for T=280 ms (and even shorter for T=260 ms), although we do not have enough data to estimate the value of $T_{c}$ nor to test whether there is truly a power law relationship. However, this would be a conceptually straightforward addition to this study that we leave as future work. We also note that similar scaling relationships can be derived for times between bursts in crisis induced intermittency (and Pommeau-Manneville intermittency), but again we do not have sufficient data to assess the validity of these relations here and leave it as potential future work.

### 4.3 Adaptation to fast pacing

In Section 3.3 we explored how the model would behave in response to a sudden increase in pacing period by setting the initial condition to approximate the state based on a pacing period of T=600 ms, and then proceeded to pace at double the frequency, i.e., with T=300 ms. When $K=1.1K_{c}$ , the model was unable to accommodate the faster pacing for approximately 1,000 beats (or approximately 300 s), i.e., propagation continued with a frequency of 1/600 ms, before giving way to a persistent epoch of turbulent behaviour. It does not appear to approach the limit cycle that we know exists from Figure 3A, while the experiments for the other values of K eventually approach their respective limit cycles, i.e., they adapt to the pacing frequency. The $K=1.1K_{c}$ experiment produces intermittency: Notice that in Figure 8B, towards the end of the simulation (from approximately 1,000 s onward) there are periods where the upper envelope gravitates around −30 mV and the lower envelope slightly above −60 mV (shown by the dashed black lines). These correspond to periods of relative order in which propagation succeeds at every beat without any major wave breaks or re-entry circuits forming, signalling motion that approaches the limit cycle, but the system always returns to chaotic behaviour. This either occurs by the upper and lower envelope moving closer together (e.g., right at the end of the simulation), corresponding to re-entrant activity^11^, or by a large drop in the lower envelope, corresponding to propagation failure and hence a domain wide descent towards the resting potential.

When $K<1.1K_{c}$ there is a transient episode of turbulent behaviour, which eventually gives way to a periodic rhythm. Compared to $K=1.1K_{c}$ , where remodelling is sparse, here the remodelled regions play a key role in sustaining propagation as they have a lower resting potential. However, the low sodium concentration present in the initial state causes a lengthened APD in the non-remodelled regions, and therefore although a wavefront generally forms upon stimulation of the domain, it faces irregular conduction blocks in the non-remodelled regions. But after a sufficient duration of pacing, the sodium concentration rises to a level as to allow for coherent propagation, and the system approaches the limit cycle that we observed in the earlier experiments. The remodelled and non-remodelled tissue therefore act in opposition to one-another in this scenario: the non-remodelled tissue has a tendency to adapt slowly and block propagation instigated at high pacing frequencies, whereas the remodelled tissue displays a short and inflexible APD, permitting propagation under a sudden increase in pacing frequency. The result of this opposition is a transient period of spatially disorganised propagation while the non-remodelled tissue adapts.

One theme throughout this work is that the experiments require simulating hundreds or thousands of beats to determine whether the behaviour is stable or not. In one sense, that is a strength in that it could connect to phenomena such as intermittency. In another it is a weakness in that it is highly computationally demanding. This would make our study difficult to adapt to experiments involving anatomically realistic atrial domains. We would argue that the primary reason that these long transients arise is the fact that the APD adapts on an ultra-slow time scale (mediated by sodium concentration). Hence, if one wished to perform similar experiments to the ones in this work on more complex domains, the most appropriate approach would be to use a significantly simpler ionic model (or a low dimensional phenomenological model like the Fenton Karma model (Fenton and Karma, 1998)) with the ultra-slow dynamics abstracted to a discrete per beat map. It is plausible that techniques that exploit separation of time scales (such as averaging and homogenisation (Pavliotis and Stuart, 2008)) could be used to derive the sodium dynamics (or equivalently a parameter that phenomenologically represents sodium) in terms of a discrete map, which could be coupled with the propagation model and updated once per beat. Moreover, due to the strong separation of time scales, it is plausible that the sodium dynamics could be accelerated without qualitatively changing the dynamics, which would allow the computational load to be reduced by an order of magnitude. The details of such an approach would require rigorous investigation that we leave as future work.

### 4.4 The role of heterogeneity

In this work we have explicitly assumed that atrial tissue is heterogeneous in that certain regions exhibit remodelled characteristics and others do not. It is notable however that real tissue can be assumed to have heterogeneities at multiple scales without necessarily assuming that it arises from a ‘pathological’ state (i.e., remodelling in this case) (Bueno-Orovio et al., 2014; Captur et al., 2017). The monodomain equations with homogeneous conduction properties can be derived using homogenisation (Colli Franzone, 2014), which essentially averages over small scales to yield a macroscopic effective conductivity. However, as we have seen with the $K=1.1K_{c}$ experiments, even a small amount of (non-uniform) contamination at microscopic scales can yield effects at the macroscopic scale. As we have seen though, the effects at the macroscopic scale are only revealed when pacing frequency is sufficiently high, and can be exacerbated by slow adaptation to an increasing pacing frequency. That is to say, the homogenised model may work perfectly well in representing the behaviour under typical conditions, but become a poor representation under specific conditions such as high frequency pacing.

One possible direction for future work would be to alter the model structure used here by relaxing the assumption of a strict ‘normal’ and ‘remodelled’ binary. It is plausible that regions may exist in different stages of remodelling, e.g., paroxysmal AF is usually associated with remodelling of calcium handling, but not with the more extensive ion-channel remodelling observed in chronic AF (Voigt et al., 2014). Another possible direction for future work would be to explore the use of a more realistic data generation process for the remodelling configurations. Here we have used the Ising model, which is an equilibrium model, but in reality an active model (e.g., a cellular Potts modelling framework like in Kudryashova et al. (2019)) would be more appropriate, since in reality the tissue itself is not a static medium. Moreover, the Ising model may not be sufficiently flexible to capture the higher-order statistical structure observed in real biological data, e.g., in Kudryashova et al. (2019), suggesting that a more complex data generation process may be required if fitting to experimental data were pursued.

The key notion conceptually in this study is not necessarily the specificity of remodelling, but rather the opposition between cells with different AP characteristics, which theoretically could arise from other factors, e.g., distribution of parasympathetic fibres (Gussak et al., 2019). One could compare this to the technique used for modelling fibrotic tissue, in which it is often the case that tissue remains conductive but not excitable (e.g., Clayton, 2018), i.e., where all ionic currents are set to 0. This would be the extreme case, where the opposition is between excitable and non-excitable regions, in which the latter can essentially operate as topological obstructions, creating fixed conduction blocks. We should note that fibrotic tissue is not always modelled as non-excitable, as recent studies have found that myofibroblasts can exhibit a fast sodium current (Koivumäki et al., 2014). Introducing these into models can provide a continuum between a regime where fibrotic regions support propagation and one where they behave more like topological obstacles (Sánchez et al., 2019). Our case is most similar to the latter scenario, where there are no fixed conduction blocks, but conduction blocks can spontaneously arise due to lack of adequate synchronization in repolarization. In this sense, what we have produced here is more similar in spirit to the classic model developed by Moe and Abildskov (1959), in which non-uniform repolarization properties is explicitly the cause of fibrillatory behaviour.

## Data Availability

The raw data supporting the conclusions of this article will be made available by the authors, without undue reservation.

## Footnotes

1 We are ignoring fibrotic regions here, and the point is that within the non-fibrotic regions the parameters are typically spatially uniform.

2 Note that after expanding out E(r) in (1), the only degrees of freedom are βK and βh , so there is always a redundancy in one of the three parameters.

3 It is the lower energy configurations that are the most likely ones, so if h>0 and the magnitude of h is sufficiently large, sites tend to significantly prefer the remodelled state. Similarly, if h<0 and |h| is sufficiently large, then sites tend to prefer the normal state.

4 Here we are referring to the idea of ‘renormalization’ and fixed points of the renormalization group (Cardy, 1996).

5 Plural because the model incorporates junctional, sarcolemmal and myoplasm sodium concentrations. When we speak of the sodium concentration without further qualification, we mean the myoplasm sodium concentration.

6 Note that period doubling refers to a bifurcation of a limit cycle where the cycle splits into two branches, see, e.g., Kuznetsov (2004). In context, one stimulus triggers a propagating wave, while the next fails, hence it takes two applications of the periodic stimulus to traverse the limit cycle.

7 When $K=1.1K_{c}$ the remodelled regions are sparse, so this is not terribly significant for the problem at hand.

8 By the symmetry of the homogeneous model, this is equivalent to propagation failure in a 1 dimensional cable, hence no spatially complex behaviour can occur.

9 Here we consider the parameters to be a spatially dependent field rather than a static vector. Therefore, an appropriate metric to measure perturbations in the parameter space would be based on, e.g., an $L^{2}$ norm, in which case it is the extent of the remodelling which determines whether the perturbation is large or small, even though the parameters for individual remodelled/non-remodelled cells differ significantly.

10 In this sense, crisis induced intermittency and Pommeau-Manneville intermittency are opposites of one another: In the former, the chaotic attractor is the primary aspect of the dynamics and is only interrupted by short bursts towards a limit cycle. In the latter, the (approximate) periodic dynamics are primary, and are only interrupted by chaotic bursts.

11 The re-entrant activity causes a portion of the domain to remain depolarised, increasing the lower envelope, but is unable to sustain as large a mass of depolarised tissue as when propagation is invoked by stimulation, lowering the upper envelope.

## References

[B1] Alonso S. Bär M. (2013). Reentry near the percolation threshold in a heterogeneous discrete model for cardiac tissue. Phys. Rev. Lett. 110, 158101. 10.1103/PhysRevLett.110.158101 25167313

[B2] Brush S. G. (1967). History of the lenz-ising model. Rev. Mod. Phys. 39, 883–893. 10.1103/RevModPhys.39.883

[B3] Bueno-Orovio A. Kay D. Grau V. Rodriguez B. Burrage K. (2014). Fractional diffusion models of cardiac electrical propagation: role of structural heterogeneity in dispersion of repolarization. J. R. Soc. Interface 11, 20140352. 10.1098/rsif.2014.0352 24920109 PMC4208367

[B4] Captur G. Karperien A. L. Hughes A. D. Francis D. P. Moon J. C. (2017). The fractal heart — embracing mathematics in the cardiology clinic. Nat. Reviews. Cardiol. 14, 56–64. 10.1038/nrcardio.2016.161 27708281 PMC5218578

[B5] Cardy J. (1996). Scaling and Renormalization in Statistical Physics. Cambridge: Cambridge University Press.

[B6] Cherry E. M. Ehrlich J. R. Nattel S. Fenton F. H. (2007). Pulmonary vein reentry–properties and size matter: insights from a computational analysis. Heart Rhythm. 4, 1553–1562. 10.1016/j.hrthm.2007.08.017 18068635

[B7] Clayton R. H. (2018). Dispersion of recovery and vulnerability to Re-entry in a model of human atrial tissue with simulated diffuse and focal patterns of fibrosis. Front. Physiology 9, 1052. 10.3389/fphys.2018.01052 30131713 PMC6090998

[B8] Colli Franzone P. (2014). Mathematical Cardiac Electrophysiology. Cham: Springer International Publishing AG.

[B9] Courtemanche M. Ramirez R. J. Nattel S. (1998). Ionic mechanisms underlying human atrial action potential properties: insights from a mathematical model. Am. J. Physiology 275, H301–H321. 10.1152/ajpheart.1998.275.1.H301 9688927

[B10] Elliott J. Mainardi L. Rodriguez Matas J. F. (2022). Cellular heterogeneity and repolarisation across the atria: an *in silico* study. Med. and Biol. Eng. and Comput. 60, 3153–3168. 10.1007/s11517-022-02640-x 36104609 PMC9537222

[B11] Fareh S. Villemaire C. Nattel S. (1998). Importance of refractoriness heterogeneity in the enhanced vulnerability to atrial fibrillation induction caused by tachycardia-induced atrial electrical remodeling. Circulation 98, 2202–2209. 10.1161/01.CIR.98.20.2202 9815876

[B12] Fenton F. Karma A. (1998). Vortex dynamics in three-dimensional continuous myocardium with fiber rotation: filament instability and fibrillation. Chaos (Woodbury, N.Y.) 8, 20–47. 10.1063/1.166311 12779708

[B13] Fortuin C. M. Kasteleyn P. W. (1972). On the random-cluster model: I. Introduction and relation to other models. Physica 57, 536–564. 10.1016/0031-8914(72)90045-6

[B14] Gallavotti G. (1999). Statistical Mechanics. Berlin, Heidelberg: Springer. 10.1007/978-3-662-03952-6

[B15] Grandi E. Pandit S. V. Voigt N. Workman A. J. Dobrev D. Jalife J. (2011). Human atrial action potential and Ca2+ model: sinus rhythm and chronic atrial fibrillation. Circulation Research 109, 1055–1066. 10.1161/CIRCRESAHA.111.253955 21921263 PMC3208665

[B16] Grebogi C. Ott E. Yorke J. A. (1983). Crises, sudden changes in chaotic attractors, and transient chaos. Phys. D. Nonlinear Phenom. 7, 181–200. 10.1016/0167-2789(83)90126-4

[B17] Grebogi C. Ott E. Romeiras F. Yorke J. A. (1987). Critical exponents for crisis-induced intermittency. Phys. Rev. A 36, 5365–5380. 10.1103/PhysRevA.36.5365 9898807

[B18] Gussak G. Pfenniger A. Wren L. Gilani M. Zhang W. Yoo S. (2019). Region-specific parasympathetic nerve remodeling in the left atrium contributes to creation of a vulnerable substrate for atrial fibrillation. JCI Insight 4 (20), e130532. 10.1172/jci.insight.130532 31503549 PMC6824299

[B19] Houben R. P. M. de Groot N. M. S. Smeets J. L. R. M. Becker A. E. Lindemans F. W. Allessie M. A. (2004). S-wave predominance of epicardial electrograms during atrial fibrillation in humans: indirect evidence for a role of the thin subepicardial layer. Heart Rhythm. 1, 639–647. 10.1016/j.hrthm.2004.08.015 15851234

[B20] Koivumäki J. T. Clark R. B. Belke D. Kondo C. Fedak P. W. M. Maleckar M. M. C. (2014). Na+ current expression in human atrial myofibroblasts: identity and functional roles. Front. Physiology 5, 275. 10.3389/fphys.2014.00275 25147525 PMC4124488

[B21] Kudryashova N. Nizamieva A. Tsvelaya V. Panfilov A. V. Agladze K. I. (2019). Self-organization of conducting pathways explains electrical wave propagation in cardiac tissues with high fraction of non-conducting cells. PLOS Comput. Biol. 15, e1006597. 10.1371/journal.pcbi.1006597 30883540 PMC6438583

[B22] Kuznetsov Y. A. (2004). Elements of Applied Bifurcation Theory. No. V. 112 in Applied Mathematical Sciences. 3rd edn. New York: Springer.

[B23] Landau L. D. Pitaevskiĭ L. P. Lifshi\t{ts} E. M. (1978). Statistical Physics (Oxford: Pergamon Press), 3d rev. edn.

[B24] Matsuda H. (1981). The ising model for population biology. Prog. Theor. Phys. 66, 1078–1080. 10.1143/PTP.66.1078

[B25] Moe G. K. Abildskov J. A. (1959). Atrial fibrillation as a self-sustaining arrhythmia independent of focal discharge. Am. Heart J. 58, 59–70. 10.1016/0002-8703(59)90274-1 13661062

[B26] Nattel S. (1999). Atrial electrophysiological remodeling caused by rapid atrial activation: underlying mechanisms and clinical relevance to atrial fibrillation. Cardiovasc. Res. 42, 298–308. 10.1016/S0008-6363(99)00022-X 10533568

[B27] Nattel S. Harada M. (2014). Atríal remodeling and atrial fibrillation. JACC 63, 2335–2345. 10.1016/j.jacc.2014.02.555 24613319

[B28] Nattel S. Burstein B. Dobrev D. (2008). Atrial remodeling and atrial fibrillation. Circulation Arrhythmia Electrophysiol. 1, 62–73. 10.1161/CIRCEP.107.754564 19808395

[B29] Pavliotis G. Stuart A. (2008). Multiscale Methohods in Applied Mathematics. New York, NY: Springer New York. 10.1007/978-0-387-73829-1

[B30] Pomeau Y. Manneville P. (1980). Intermittent transition to turbulence in dissipative dynamical systems. Commun. Math. Phys. 74, 189–197. 10.1007/BF01197757

[B31] Ramdat Misier A. R. Opthof T. van Hemel N. M. Defauw J. J. de Bakker J. M. Janse M. J. (1992). Increased dispersion of “refractoriness” in patients with idiopathic paroxysmal atrial fibrillation. JACC 19, 1531–1535. 10.1016/0735-1097(92)90614-S 1593049

[B32] Sánchez J. Gomez J. F. Martinez-Mateu L. Romero L. Saiz J. Trenor B. (2019). Heterogeneous effects of fibroblast-myocyte coupling in different regions of the human atria under conditions of atrial fibrillation. Front. Physiology 10, 847. 10.3389/fphys.2019.00847 31333496 PMC6620707

[B33] Swendsen R. H. Wang J.-S. (1987). Nonuniversal critical dynamics in monte carlo simulations. Phys. Rev. Lett. 58, 86–88. 10.1103/PhysRevLett.58.86 10034599

[B34] Ten Tusscher K. H. W. J. Panfilov A. V. (2003). Reentry in heterogeneous cardiac tissue described by the luo-rudy ventricular action potential model. Am. J. Physiology-Heart Circulatory Physiology 284, H542–H548. 10.1152/ajpheart.00608.2002 12388228

[B35] van Schie M. S. Talib S. Knops P. Taverne Y. J. H. J. de Groot N. M. S. (2024). Conduction velocity and anisotropic properties of fibrillation waves during acutely induced and long-standing persistent AF. JACC Clin. Electrophysiol. 10, 1592–1604. 10.1016/j.jacep.2024.02.001 38752952

[B36] Varela M. Colman M. A. Hancox J. C. Aslanidi O. V. (2016). Atrial heterogeneity generates Re-entrant substrate during atrial fibrillation and anti-arrhythmic drug action: mechanistic insights from canine atrial models. PLOS Comput. Biol. 12, e1005245. 10.1371/journal.pcbi.1005245 27984585 PMC5161306

[B37] Vigmond E. Pashaei A. Amraoui S. Cochet H. Hassaguerre M. (2016). Percolation as a mechanism to explain atrial fractionated electrograms and reentry in a fibrosis model based on imaging data. Heart Rhythm. 13, 1536–1543. 10.1016/j.hrthm.2016.03.019 26976038

[B38] Voigt N. Heijman J. Trausch A. Mintert-Jancke E. Pott L. Ravens U. (2013). Impaired na+-Dependent regulation of acetylcholine-activated inward-rectifier K+ current modulates action potential rate dependence in patients with chronic atrial fibrillation. J. Mol. Cell. Cardiol. 61, 142–152. 10.1016/j.yjmcc.2013.03.011 23531443

[B39] Voigt N. Heijman J. Wang Q. Chiang D. Y. Li N. Karck M. (2014). Cellular and molecular mechanisms of atrial arrhythmogenesis in patients with paroxysmal atrial fibrillation. Circulation 129, 145–156. 10.1161/CIRCULATIONAHA.113.006641 24249718 PMC4342412

[B40] Wijffels M. C. Kirchhof C. J. Dorland R. Allessie M. A. (1995). Atrial fibrillation begets atrial fibrillation. Circulation 92, 1954–1968. 10.1161/01.CIR.92.7.1954 7671380

[B41] Xie F. Qu Z. Garfinkel A. Weiss J. N. (2001). Electrophysiological heterogeneity and stability of reentry in simulated cardiac tissue. Am. J. Physiology. Heart Circulatory Physiology 280, H535–H545. 10.1152/ajpheart.2001.280.2.H535 11158949

