# Supplementary material for: Spatially heterogeneous ionic remodelling promotes wavefront instability in simulated atrial tissue
Source: Front Netw Physiol. 2026 Jul 30;6:1849952. doi: 10.3389/fnetp.2026.1849952 (PMC13467856; doi:10.3389/fnetp.2026.1849952)
Supplement: Supplementary file 1 [file DataSheet1.pdf]

Supplementary Material

1 SUPPLEMENTARY DATA

1.1 Effect of Sodium

The top row of Figure S1 demonstrates the effect of sodium concentration in the case of a cell with non-remodelled parameters paced every $T = 300$ ms, and the effect of altering the sodium concentration based on pacing at $T = 450$ ms and $T = 600$ ms. The sodium concentration $[\mathrm{Na}]_\mathrm{i}$ decreases as T increases, and consequently the sodium-calcium exchanger and sodium-potassium pump transients are altered in such a way as to prolong the duration of repolarization (right of Figure S1). Hence, for high pacing frequencies, if the sodium concentration is too low, the inactivation gates of I_{Na} can fail to recover before the next stimulus (middle of Figure S1), causing wave propagation to fail. The bottom row of Figure S1 shows the same effect but with the cAF parameters, and demonstrates that the effect is less pronounced, and the I_{Na} gating variables recover to a much greater degree in this case.

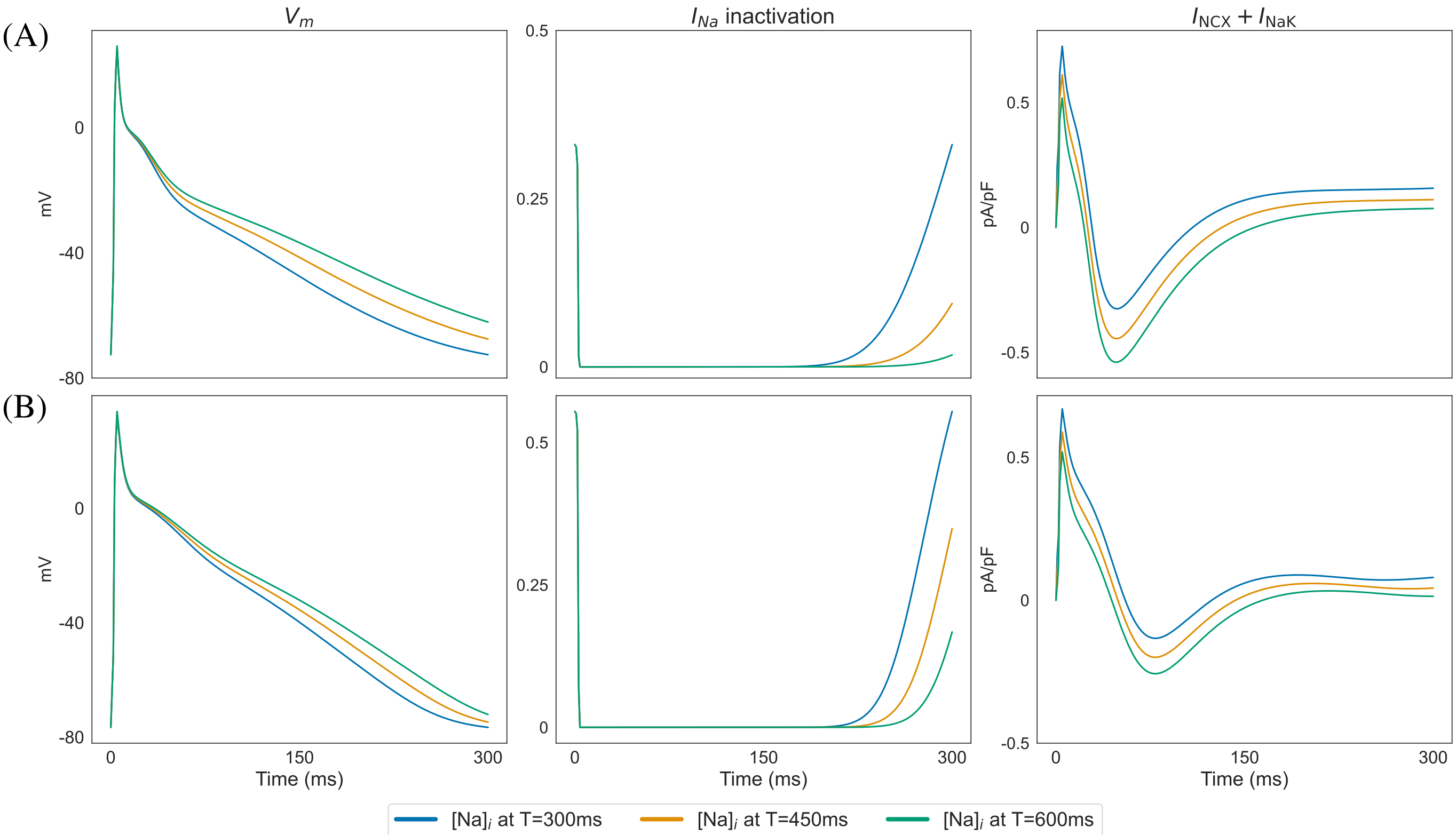

Figure S1. The effect of varying the initial sodium concentrations on the non-remodelled **(A)** and remodelled **(B)** single cell model paced every 300 ms. The blue realisation is on the limit cycle for pacing $T = 300$ ms, while the other two have the same initial state except that the sodium concentrations are adjusted to those on the limit cycle for $T = 450$ ms and $T = 600$ ms. The left shows the effect on the action potential, the middle shows the product of the two I_{Na} inactivation gate variables hj, and the right shows the effect on the sum of the sodium calcium exchanger and sodium potassium pump.

1.2 Numerical Methods

To approximate the diffusion term, we use a finite volume method, which is advantageous over a typical finite differences method due to the discontinuities in the diffusivity. Essentially, a finite volume method enforces continuity in the flux terms

$$F^x(x,y) = D_x(x,y)\frac{\partial V_m(x,y)}{\partial x}$$
$$F^y(x,y) = D_y(x,y)\frac{\partial V_m(x,y)}{\partial y},$$

even with discontinuous diffusivity coefficients D_x and D_y. If one assumes continuity at each lattice point, then approximations of the flux at the half spaces on the lattice can be worked out in terms of a harmonic average of the diffusivities:

$$F^x_{i+\frac{1}{2},j} \equiv \frac{2[D_x]_{i+1,j}[D_x]_{i,j}}{[D_x]_{i+1,j}+[D_x]_{i,j}}\frac{[V_m]_{i+1,j}-[V_m]_{i,j}}{\Delta x}$$
$$F^y_{i,j+\frac{1}{2}} \equiv \frac{2[D_y]_{i,j+1}[D_y]_{i,j}}{[D_y]_{i,j+1}+[D_y]_{i,j}}\frac{[V_m]_{i,j+1}-[V_m]_{i,j}}{\Delta y},$$

and then the diffusion term is approximated as

$$\left[\frac{\partial}{\partial x}\left(D_x\frac{\partial V_m}{\partial x}\right)+\frac{\partial}{\partial y}\left(D_y\frac{\partial V_m}{\partial y}\right)\right]_{i,j} \approx \frac{F^x_{i+\frac{1}{2},j}-F^x_{i-\frac{1}{2},j}}{\Delta x}+\frac{F^y_{i,j+\frac{1}{2}}-F^y_{i,j-\frac{1}{2}}}{\Delta y}.$$

The integrator we have used is the operator splitting method given in Qu and Garfinkel (1999).

2 SUPPLEMENTARY TABLES AND FIGURES

2.1 Figures

REFERENCES

Qu, Z. and Garfinkel, A. (1999). An advanced algorithm for solving partial differential equation in cardiac conduction. *IEEE Transactions on Biomedical Engineering* 46, 1166–1168. doi:10.1109/10.784149

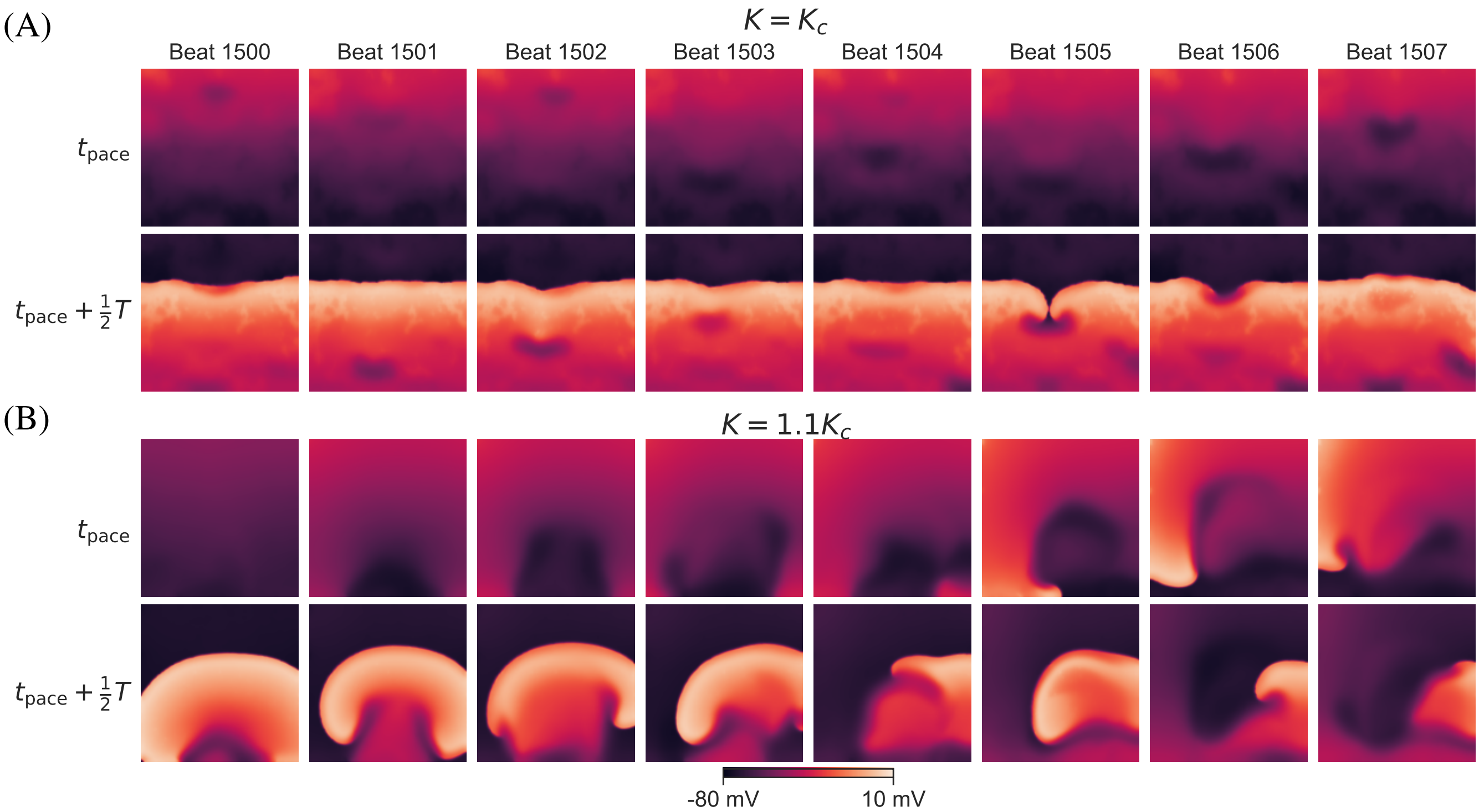

Figure S2. **(A)**: Snapshots of 8 beats for $T = 290$ ms and $K = K_c$. An example of a small wave break in this experiment can be seen on beat 1505, and the start of another one on beat 1506. **(B)**: the same beats for $T = 290$ ms and $K = 1.1K_c$. The behaviour in this case is much more chaotic, and potential re-entrant circuits can be seen.

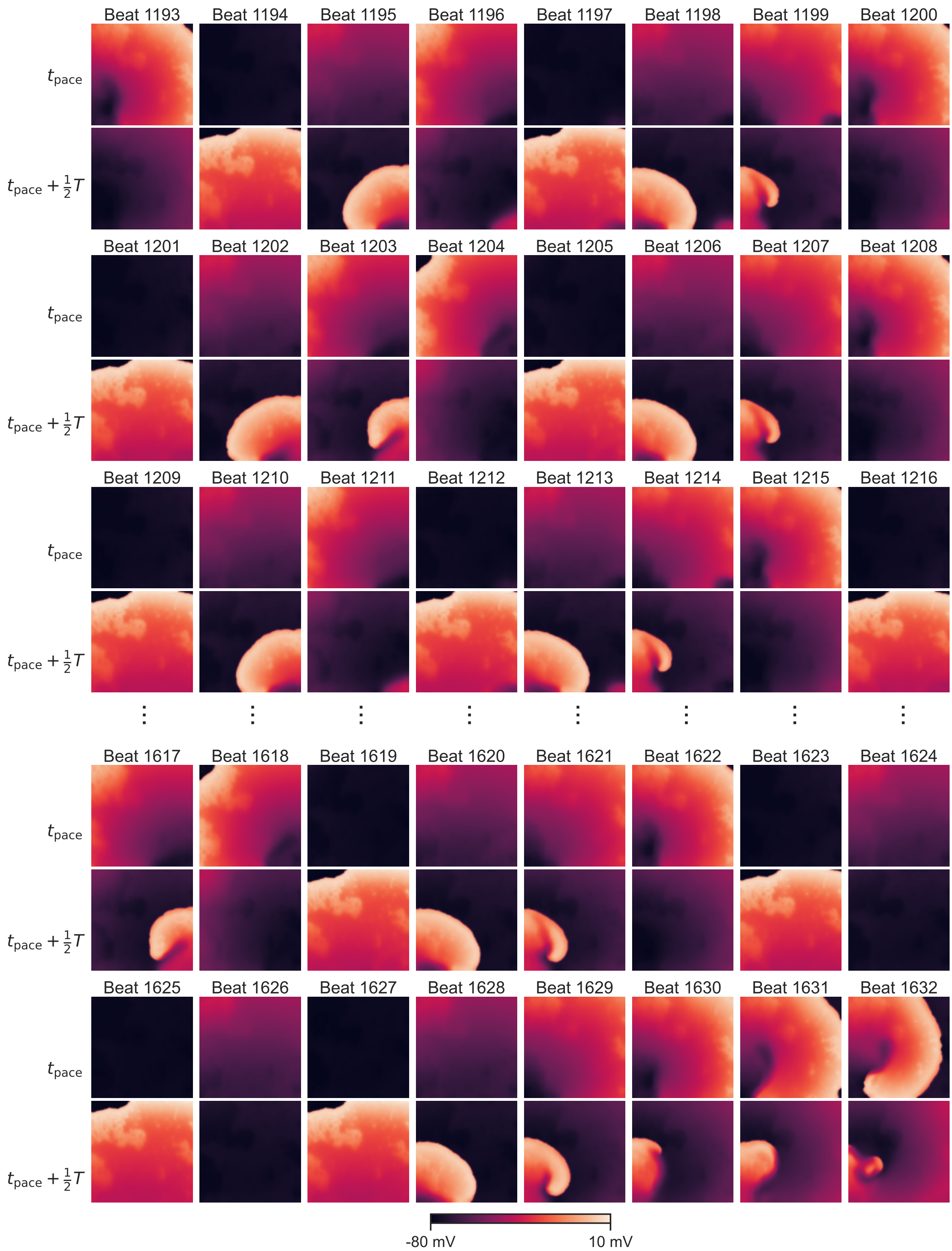

Figure S3. Snapshots of period 8 behaviour. The top block (beat 1193-1200) shows the 8 beats in the cycle. The next two blocks (beats 1201-1216) show the beats immediately after falling off the limit cycle. Clearly similar behaviour persists, but there is a shift in the phase of the beats. The bottom two blocks (beats 1617-1632) shows that the same 8 beats (with occasional phase shifts) continue up until around beat 1630, where the behaviour becomes more complex.

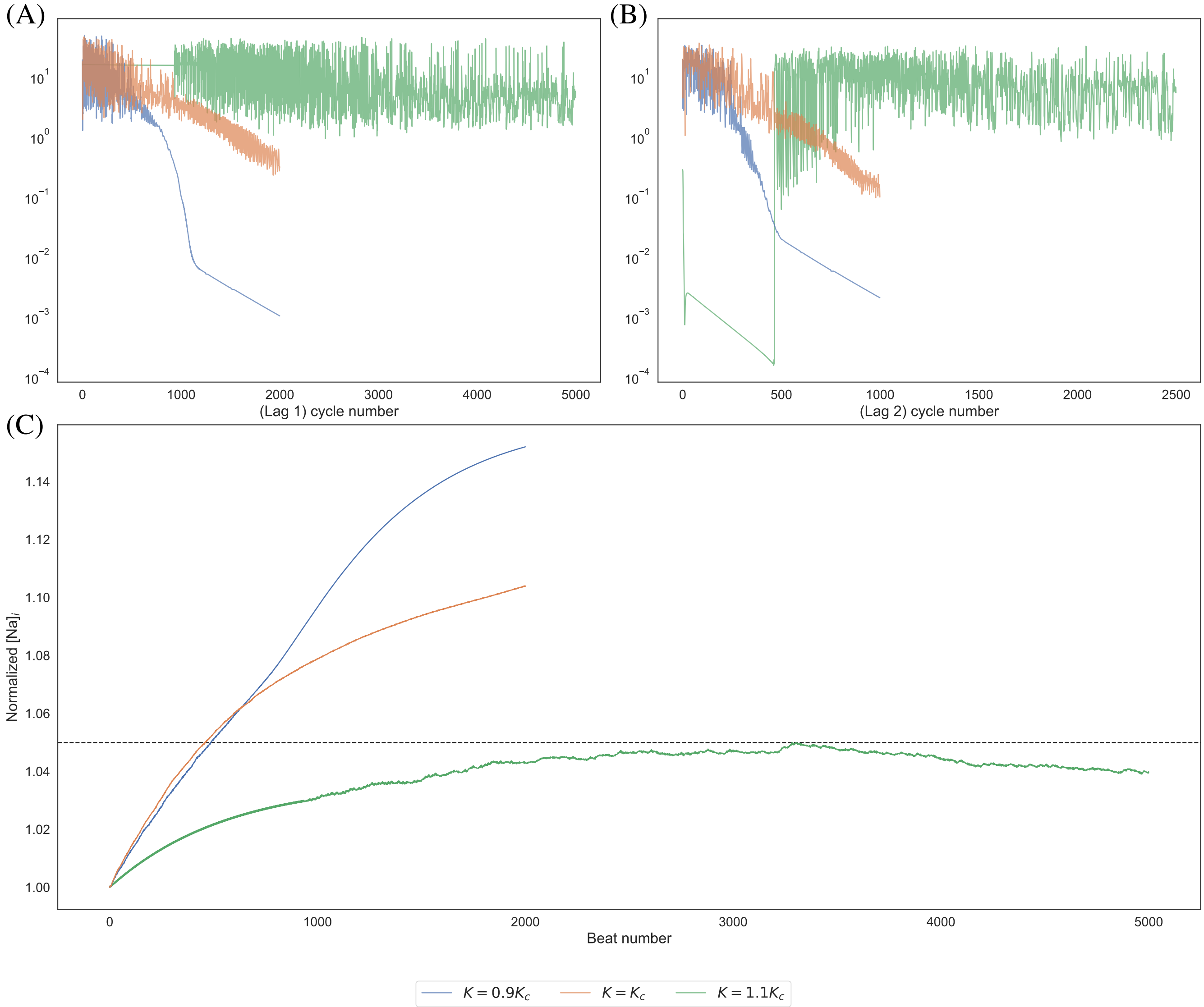

Figure S4. **(A)**: $d^{(1)}$ values for $T = 300$ ms in the case of the altered initial condition. **(B)**: Same but for $d^{(2)}$. It can be seen that when $K = 1.1K_c$, every other beat fails to propagate for approximately 1000 beats, followed by a sharp transition to turbulent behaviour. **(C)**: $[\mathrm{Na}]_i$ averaged over the bottom 5% of the domain (in the bottom 20 rows of the grid, near the paced edge) relative to the initial concentration. For $K = 0.9K_c$ and $K = K_c$, the growth is similar to what we saw in the main manuscript, while for $K = 1.1K_c$, the growth is initially faster than the rest of the domain, but plateaus and begins to decrease beyond about 3500 beats.

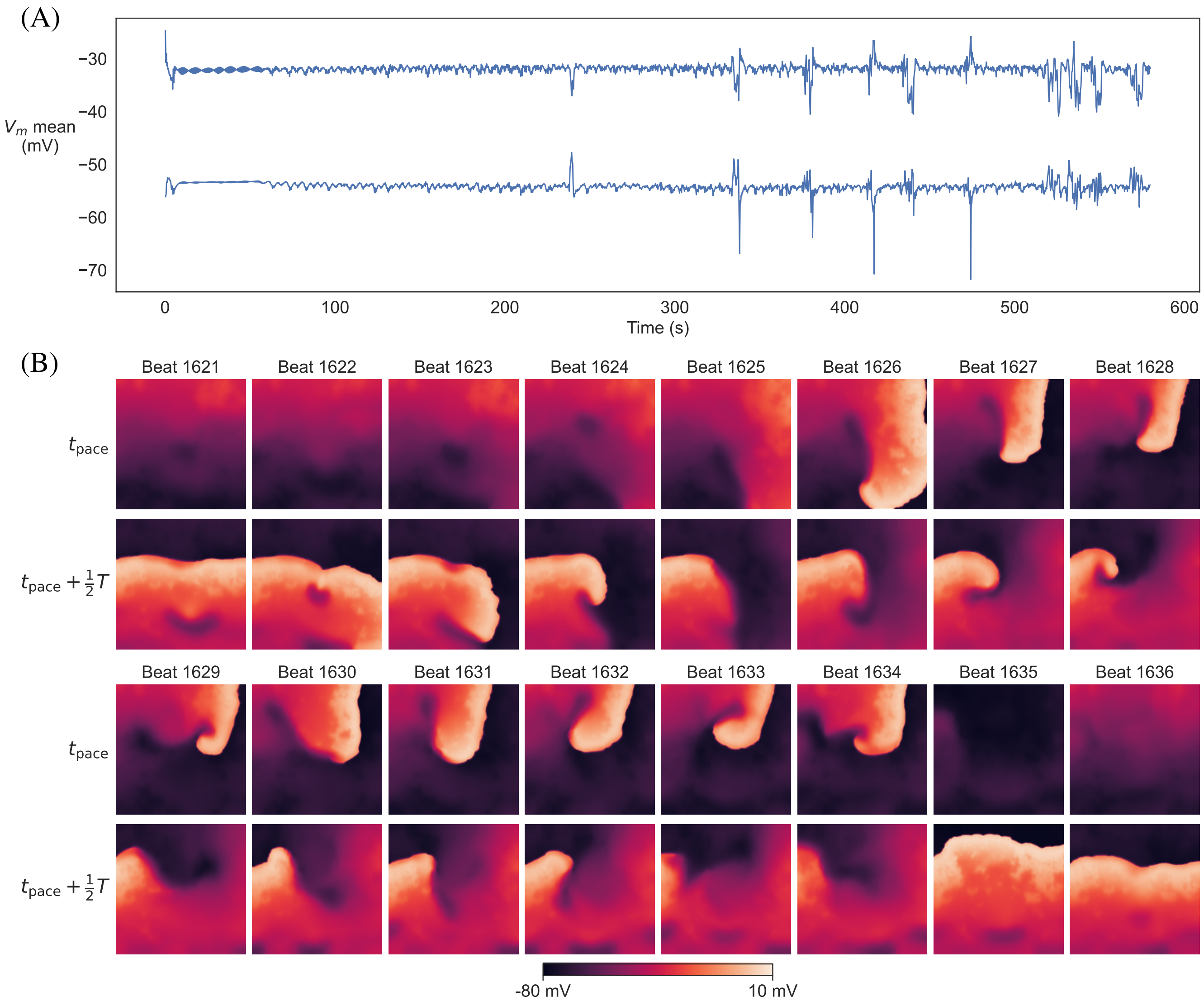

Figure S5. **(A)**: The envelope of V_m spatial average for a different realisation of $K = K_c$ and $T = 290$ ms. The typical behaviour is similar to that of Figure S2A (i.e. minor wave breaks but generally coherent propagation), evidenced by the typically small fluctuations in the envelope, however intermittent episodes of more irregular behaviour followed by propagation failure occur, evidenced by the large spikes in the envelope. **(B)**: Example of AP snapshots near the spike that occurs at 475 seconds above. We can see that the wavefront begins in a similar state to that of Figure S2A, but is subsequently blocked on the right hand side of the domain, creating a pathway for re-entry. After 12 beats, the re-entrant circuit is blocked and propagation returns to normal.
